# Venom composition and pain-causing toxins of the Australian great carpenter bee *Xylocopa aruana*

**DOI:** 10.1038/s41598-022-26867-8

**Published:** 2022-12-22

**Authors:** Naiqi Shi, Tibor G. Szanto, Jia He, Christina I. Schroeder, Andrew A. Walker, Jennifer R. Deuis, Irina Vetter, György Panyi, Glenn F. King, Samuel D. Robinson

**Affiliations:** 1 Institute for Molecular Bioscience, The University of Queensland, Brisbane, QLD 4072 Australia; 2 Department of Biophysics and Cell Biology, Faculty of Medicine, University of Debrecen, 4032 Debrecen, Hungary; 3 School of Pharmacy, The University of Queensland, Brisbane, QLD 4102 Australia

**Keywords:** Peptides, Proteomics, Entomology

## Abstract

Most species of bee are capable of delivering a defensive sting which is often painful. A solitary lifestyle is the ancestral state of bees and most extant species are solitary, but information on bee venoms comes predominantly from studies on eusocial species. In this study we investigated the venom composition of the Australian great carpenter bee, *Xylocopa aruana* Ritsema, 1876. We show that the venom is relatively simple, composed mainly of one small amphipathic peptide (XYTX_1_-Xa1a), with lesser amounts of an apamin homologue (XYTX_2_-Xa2a) and a venom phospholipase-A_2_ (PLA_2_). XYTX_1_-Xa1a is homologous to, and shares a similar mode-of-action to melittin and the bombilitins, the major components of the venoms of the eusocial *Apis mellifera* (Western honeybee) and *Bombus* spp. (bumblebee), respectively. XYTX_1_-Xa1a and melittin directly activate mammalian sensory neurons and cause spontaneous pain behaviours in vivo, effects which are potentiated in the presence of venom PLA_2_. The apamin-like peptide XYTX_2_-Xa2a was a relatively weak blocker of small conductance calcium-activated potassium (K_Ca_) channels and, like *A. mellifera* apamin and mast cell-degranulating peptide, did not contribute to pain behaviours in mice. While the composition and mode-of-action of the venom of *X. aruana* are similar to that of *A. mellifera*, the greater potency, on mammalian sensory neurons, of the major pain-causing component in *A. mellifera* venom may represent an adaptation to the distinct defensive pressures on eusocial Apidae.

## Introduction

There are approximately 20,000 species of bees (Hymenoptera: clade Anthophila). Of the seven currently recognised bee families, Apidae is the most diverse (> 5000 species) and includes the well-known eusocial honeybees (genus *Apis*), bumble bees (genus *Bombus*) and stingless bees (Tribe Meliponini) as well as the carpenter bees (genus *Xylocopa*) of which some species exhibit social tendencies under certain conditions^1^ but none are eusocial^2^. All Apidae, both solitary and eusocial (with the exception of the stingless bees), are capable of delivering a defensive sting^3^.

By far the most studied bee venom is that of the Western honeybee *Apis mellifera*. Its venom is dominated by a 26-residue cysteine-free amphipathic peptide known as melittin which makes up 40–60% of the dry weight of the venom^4^. Another major components is venom phospholipase-A_2_ (PLA_2_; 10–12% dry weight), with smaller amounts of histamine (5–10%), venom hyaluronidase (1–2%) and the small disulfide-rich peptides apamin (3%) and mast cell-degranulating (MCD)-peptide (2%)^4^. Other reported components are present in trace amounts. Melittin, which can form pores in cell membranes^5,6^, is thought to be the major pain-causing component of honeybee venom^7^, while the role of other venom components in the context of defence or pain is not well-established.

Bumble bee venoms have also received some attention. The major components of their venoms are bombolitins, 17–19 residue peptides which like melittin are cysteine-free and amphipathic^8,9^. Other venom components include a so-called MCD-peptide^10^, which is structurally and functionally related to its namesake in honeybee venom, and venom PLA_2_. Bombus venom components have not been studied directly in the context of defence or pain, but the apparent general similarity in venom composition to honeybee venom is suggestive of a similar mode-of-action.

Investigations on the venoms of solitary Apidae are comparatively limited. A study on the venom of the cleptoparasitic mourning bee *Melecta albifrons*, reported an 18-residue cysteine-free amphipathic peptide called melectin^11^, which is the major component of the venom and appears to share similar activity to melittin and the bombolitins. The major components of the venom of the Japanese carpenter bee *Xylocopa appendiculata* are two 17-residue cysteine-free and amphipathic peptides, called xylopin (Xac-1) and xylopinin (Xac-2), and a venom phospholipase-A_2_^12,13^. Finally, a recent study of the venom of *Xylocopa violacea* revealed that it is largely composed of melittin/xylopin-like peptides, apamin-like peptides and phospholipase-A_2_^14^.

Venom composition and venom toxin bioactivity, like other phenotypic traits, are frequently adapted to the specific ecological niches occupied by the organisms that produce them. For example, certain cone snail and snake venom toxins show taxon-specific toxicity towards the natural prey items of these animals^15,16^, and true bug (Hemiptera) venom composition evolved rapidly in response to transitions from predation and haematophagy^17^. The Apidae use their venoms solely for defence, but solitary and eusocial Hymenoptera are faced with distinct defensive selection pressures. Solitary species sting solely in self-defence, while eusocial species sting for both self-defence and defence of their colony’s brood and food stores, including against large vertebrate predators. Accordingly, it has been hypothesized, that in Hymenoptera, venom composition may have co-evolved with lifestyle^18,19^. However differences in venom composition and pharmacology between solitary and eusocial Apidae have not been directly examined.

Here, we performed a detailed analysis of the venom composition, pharmacology and pain-causing mechanism(s) of the carpenter bee *Xylocopa aruana*, and compared it to that of the eusocial honey bee *A. mellifera*. We found that the venom of *X. aruana* is relatively simple and similar to that of *A. mellifera* and induces pain via a similar mode-of-action. The major pain-casing agent in *A. mellifera* venom, melittin, was more potent than its *X. aruana* counterpart in activating mammalian sensory neurons, and may represent an adaptation to the distinct defensive pressures on eusocial Apidae.

## Results

### The venom of *Xylocopa aruana* is simple in composition and similar to that of *Apis mellifera*

**Figure 1:**
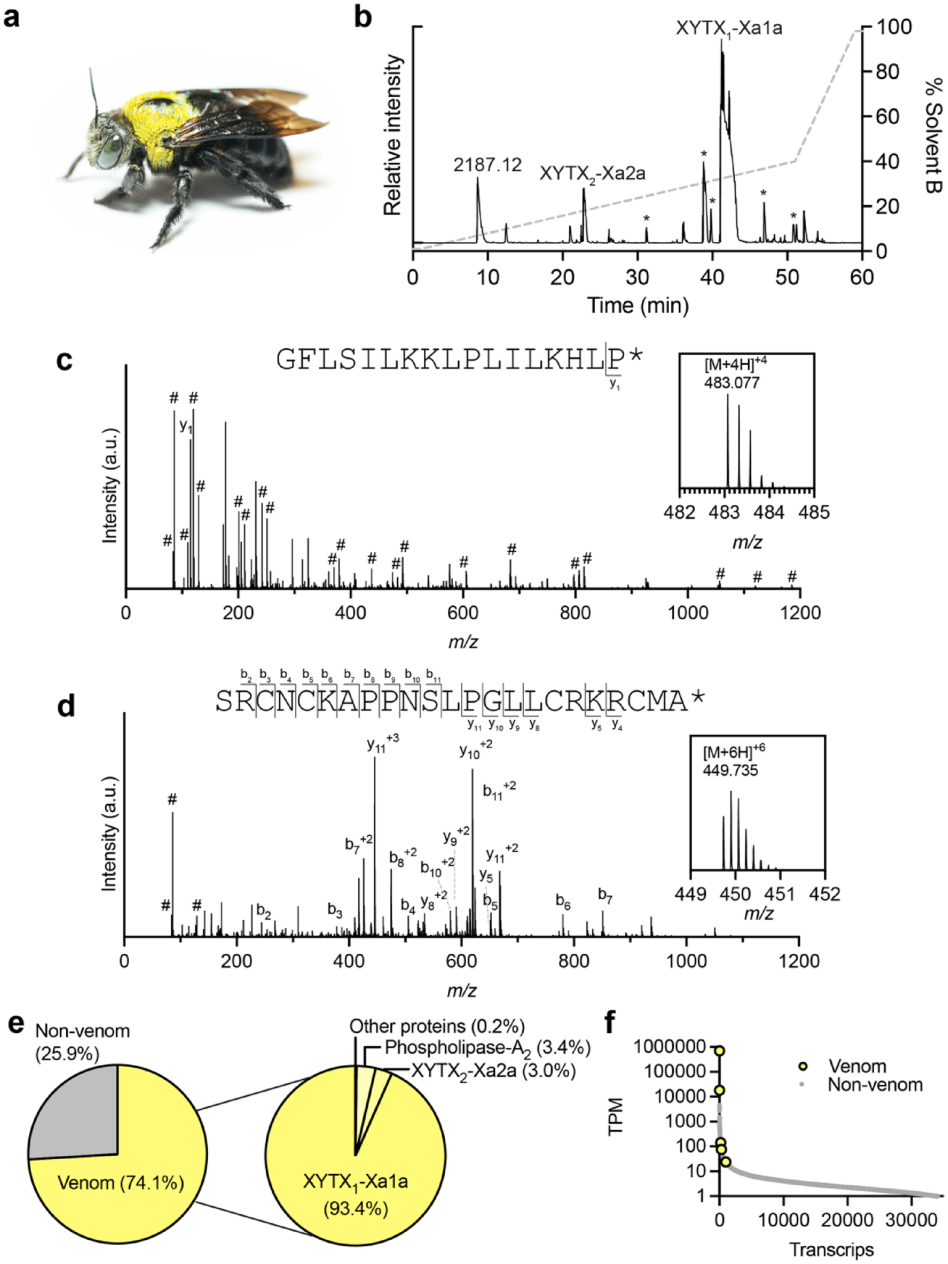
The venom composition of *X. aruana*. (**a**) Adult female *X. aruana* (the specimen that was used in this study). (**b**) Total ion chromatogram from LC–MS/MS analysis of *X. aruana* venom with peaks corresponding to identified venom peptides labelled. Additional proteins were detected in the reduced, alkylated and trypsin-digested venom sample. The signal corresponding to XYTX_1_-Xa1a is saturated. Asterisks mark derivatives of XYTX_1_-Xa1a. (**c**) Complete CID-MS/MS spectrum of the [M + 4H]^+4^ precursor ion of XYTX_1_-Xa1a. Inset: [M + 4H]^+4^ MS spectrum (theoretical [M + 4H]^+4^ = 483.075). (**d**) Complete CID-MS/MS spectrum of the [M + 6H]^+6^ precursor ion of XYTX_2_-Xa2a (in the reduced and alkylated venom sample). Inset: [M + 6H]^+6^ MS spectrum (theoretical [M + 6H]^+6^ = 449.734). b-, y- and internal ions (#) are labelled (for clarity, other ions are not labelled). Inset: MS spectrum. (e) Venom component-encoding transcripts (i.e., those encoding peptides detected in the venom by MS) comprised 74.1% of total transcript expression. Of these, transcripts encoding XYTX_1_-Xa1a, XYTX_2_-Xa2a, PLA_2_, and other venom proteins comprised 93.4, 3.0, 3.4, and 0.2%, respectively, of venom component expression. (**f**) Venom component-encoding transcripts (highlighted in yellow) are found exclusively in the highly expressed portion of the venom apparatus transcriptome. *TPM* transcripts per million.

We used a combined transcriptomic and mass spectrometry (MS)-based strategy to generate a full profile of the composition of polypeptides in venom from an individual adult female *X. aruana* (Fig. 1a). RNA extracted from the venom glands was used to generate a venom gland transcriptome. We obtained 24,904,864 demultiplexed paired-end reads from Illumina NextSeq RNA sequencing, which, following adaptor trimming, quality trimming and filtering and error correction, were assembled de novo using Trinity to yield a total of 43,374 contigs. Venom was collected by squeezing of the contents of the venom reservoir and venom duct into water. Liquid chromatography-tandem MS (LC–MS/MS) data from three venom samples (native; reduced and alkylated; reduced, alkylated, and trypsin-digested) were searched against a database comprising the translated venom gland transcriptome.

Analysis of the venom of *X. aruana* by LC–MS indicated that it was relatively simple (Fig. 1b). By top-down sequencing of the native and reduced and alkylated venom samples we identified two peptides (Fig. 1b–d, Table 1). A 17 amino acid, cysteine-free peptide with an amidated C-terminus, which we called XYTX_1_-Xa1a, dominated the venom. The total ion chromatogram of the native venom, shown in Fig. 1b, illustrates the relative abundance of this peptide. Numerous derivatives (e.g. truncated version of the peptide) were also detected, although at much lower abundance, and are labelled with asterisks in Fig. 1b. We cannot confirm whether these derivatives are present in the natural venom or are an artefact of our venom collection technique. In the venom gland transcriptome two near-identical transcripts (probably representing either allelic variants or paralogues) encoded the mature peptide XYTX_1_-Xa1a, differing only by synonymous substitutions at two sites. Together, these accounted for 93.4% of venom component expression (Fig. 1e,f). The second peptide was 23 amino acids in length with four cysteine residues and an amidated C-terminus. A peak with mass [M + 4H]^4+^  = 629.061 (theoretical [M + 4H]^4+^  = 629.063) and MS/MS spectra corresponding to the monomeric peptide was detected in the native venom sample. No peaks with a mass corresponding to that of the dimeric peptide were detected, indicating that this peptide exists in the venom as a monomer and not as a dimer. This peptide, which we called XYTX_2_-Xa2a, accounted for 3.0% of venom component expression. Analysis of the native venom sample by matrix-assisted laser desorption/ionization-time of flight (MALDI-TOF) MS was consistent with the data obtained by LC–ESI–MS i.e. Four major peaks corresponded to XYTX_1_-Xa1a (and two derivatives) and XYTX_2_-Xa2a (Fig. S2).

**Table 1:** Venom components of *X. aruana*.

	TPM	Sequence
**Major venom components**		
XYTX_1_-Xa1a	691,980^†^	GFLSILKKLPLILKHLP*
XYTX_2_-Xa2a	22,179	SRCNCKAPPNSLPGLLCRKRCMA*
Phospholipase A_2_^a^	24,834	
**Other trace components**		
Hyaluronidase^a^	451	
Icarapin^a^	306	
Serine carboxypeptidase^a^	166	
Peptidyl-prolyl cis–trans isomerase^a^	143	
DPP-4^a^	76	
Serine protease^a^	72	

*C-terminal amidation.

^†^The TPM value is the sum of two paralogous transcripts encoding the same mature peptide.

^a^Detected in reduced, alkylated, trypsin-digested venom sample; sequence not shown. A non-curated summary of the bottom-up sequencing results is included as Supplementary Dataset 1.

**Figure 2:**
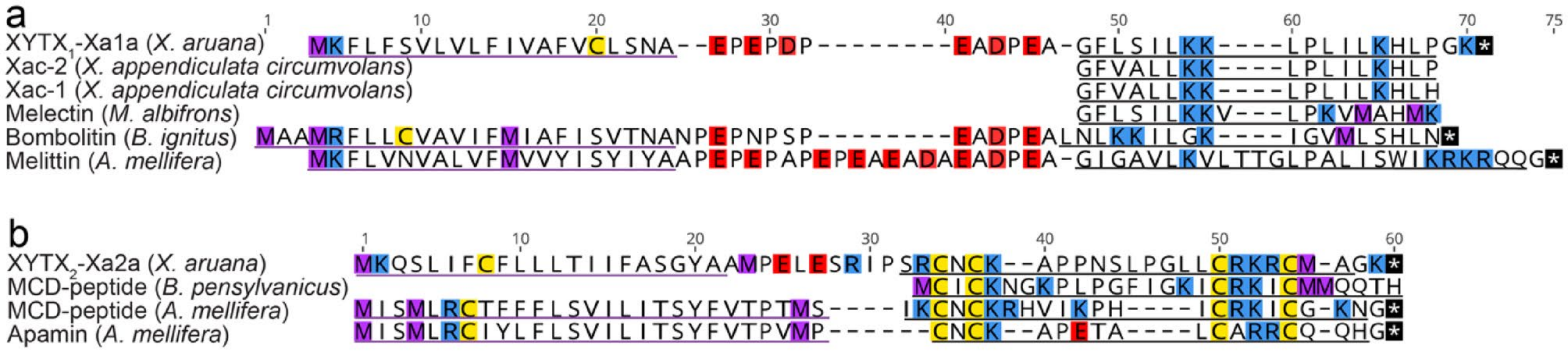
Amino acid sequence alignments of the peptide toxins of *X. aruana* venom with homologues from other Apidae venoms. (**a**) XYTX_1_-Xa1a alignment with Xac-1 (Uniprot: C0HKQ5), Xac-2 (Uniprot: C0HKQ6), melectin (Uniprot: P86170), bombolitin (Uniprot: D0VDZ4) and melittin (Uniprot: P01501). (**b**) XYTX_2_-Xa2a alignment with apamin (Uniprot: P01500), *Bombus* MCD-peptide (Uniprot: P04567) and *Apis* MCD-peptide (Uniprot: P01499). Methionine, lysine/arginine, aspartate/glutamate, and cysteine residues and stop codons are highlighted in purple, blue, red, yellow and black, respectively. Signal peptides and mature peptides are underlined in purple and grey, respectively. Post-translational modifications are not shown.

We used the BLASTp algorithm to search the UniProt/GenBank protein database for sequences related to XYTX_1_-Xa1a and XYTX_2_-Xa2a. XYTX_1_-Xa1a was similar to Xac-1 (Uniprot: C0HKQ5; 13/17 residues) and Xac-2 (Uniprot: C0HKQ6; 14/17 residues) from the venom of *X. appendiculata* (Fig. 2a)^13^. Alignment of the precursor sequence of XYTX_1_-Xa1a with those of bombolitin and melittin were indicative of homology i.e. common ancestry (see Fig. 2a). No significant alignments were detected for XYTX_2_-Xa2a, although we noted general similarity (i.e. in length and presence of four cysteine residues) to the *Bombus* MCD-peptide (Uniprot: P04567), *Apis* MCD-peptide (Uniprot: P01499) and apamin (Uniprot: P01500). Alignment of the prepropeptide sequence of XYTX_2_-Xa2a with those of the MCD-peptides and apamin were suggestive of homology (see Fig. 2b).

Several other proteins were detected by bottom-up sequencing of the reduced, alkylated and trypsin-digested venom sample. Of these, a PLA_2_ was the most highly expressed (3.4% of venom component expression) (Fig. 1c). This was almost identical (96% amino acid identity) to that reported in the venom of *X. appendiculata* (Uniprot: I7GQA7) and similar to those reported from *Bombus* and *Apis* venoms (Fig. S1). The remaining proteins were expressed at much lower levels, together constituting only 0.2% of venom component expression (Fig. 1c, Dataset 1). These proteins include some which have been implicated as toxins e.g. hyaluronidase, others likely serve a role in the production and/or maturation of the peptide toxins e.g. dipeptidyl peptidase-4 (DPP-4), while others appear to be endogenous proteins e.g. actin. A non-curated summary of the bottom-up sequencing results is included as Supplementary Dataset 1.

The high proportion of venom gland-derived reads encoding the two peptides XYTX_1_-Xa1a and XYTX_2_-Xa2a and the venom PLA_2_, as well as the assignment of the major peaks of the total ion chromatogram of the native venom, strongly suggested that together these three polypeptides represent the major components of *X. aruana* venom (Fig. 1).

### Pharmacological activity of *X. aruana* venom peptides

We prepared XYTX_1_-Xa1a and XYTX_2_-Xa2a by solid phase peptide synthesis (SPPS). Oxidative folding of the linear XYTX_2_-Xa2a produced a single major peak which eluted at the same retention time as the native peptide in the venom (Fig. S3).

**Figure 3:**
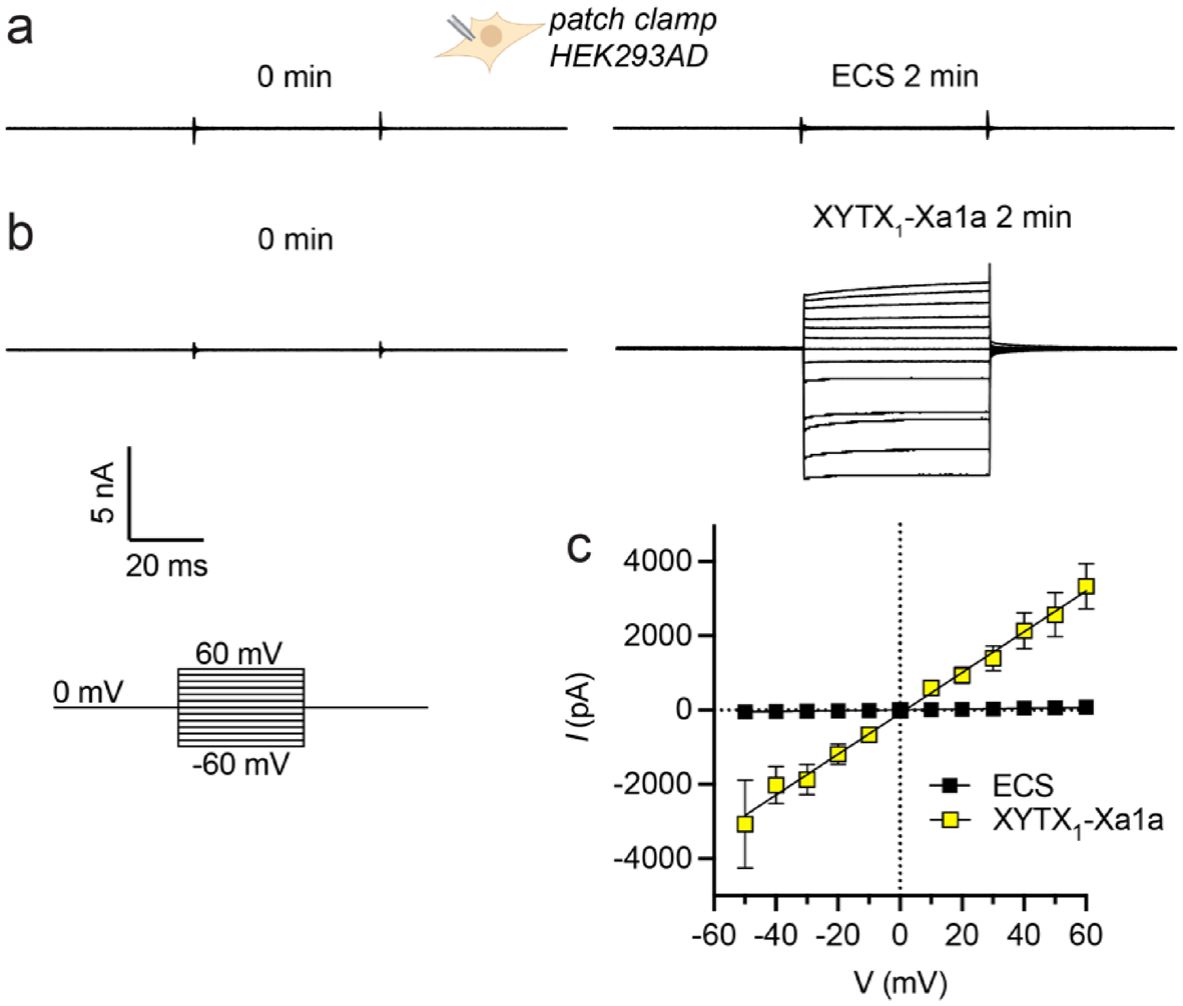
XYTX_1_-Xa1a causes a leak current in HEK293AD cells. (**a**) Representative current traces in HEK293AD cells at voltages of − 60 to + 60 mV (10 mV steps) acquired before application and 2 min after application of (**a**) extracellular solution (ECS) or (**b**) 30 μM XYTX_1_-Xa1a. Scale bar for panels a-b and voltage protocol used to investigate leak current are shown on the bottom left. (**c**) Current–voltage (I–V) relationship 2 min after addition of ECS (black) or XYTX_1_-Xa1a (30 μM; yellow). Data are expressed as mean ± SEM (*n* = 5–6 cells) and fitted to a simple linear regression. Equivalent data for melittin are shown in supplemental information Fig. S2.

The mature peptide of XYTX_1_-Xa1a shares a similar primary structure with Xac-1, Xac-2 and melectin. Previous studies of these peptides have indicated amphipathic α-helical structure in membrane-mimicking solvents, degranulation of mast cells, and antimicrobial activity, all of which are consistent with a mode-of-action involving disruption of cell membranes^11–13^. We hypothesised that XYTX_1_-Xa1a would share the same activity. We tested, by whole-cell patch-clamp electrophysiology, the capacity of XYTX_1_-Xa1a to directly induce leak currents in cell membranes. For these experiments we used HEK293AD cells, which lack appreciable expression of the ion channels found in neurons. At 4 min after application of XYTX_1_-Xa1a (30 μM) we recorded leak currents at test potentials ranging from − 60 to + 60 mV in 10-mV increments from a holding potential of 0 mV every 6 s. At + 60 mV, we recorded currents of 3.3 ± 0.6 nA (mean ± SEM, *n* = 6 cells) compared with 0.08 ± 0.02 nA (*n* = 5 cells) for time-matched negative controls (application of extracellular solution (ECS)) (Fig. 3a–c). These data are consistent with a membrane disrupting mode of action for XYTX_1_-Xa1a, similar to that of melittin (Fig. S4).

**Figure 4:**
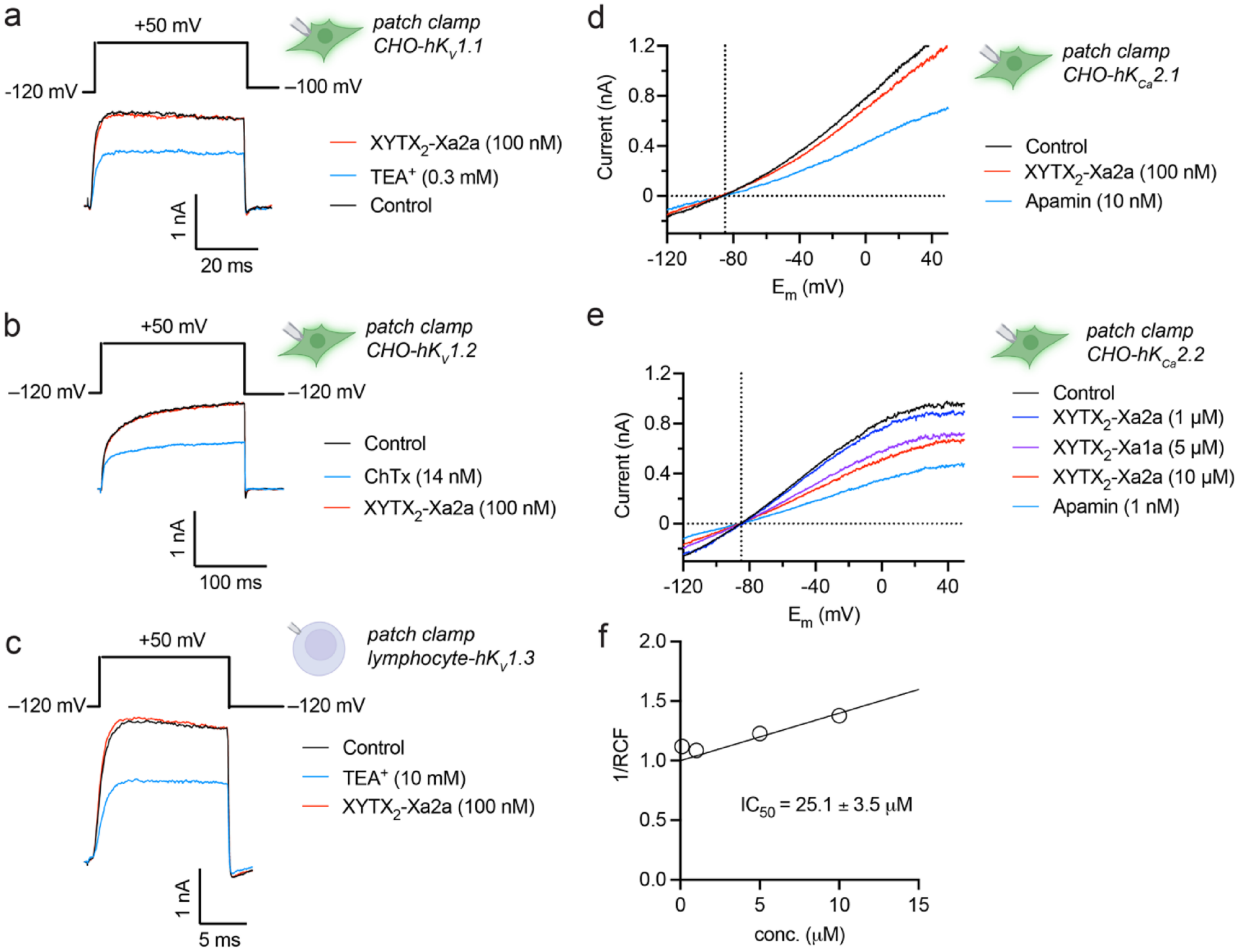
Activity of XYTX_2_-Xa2a on hK_V_1.1, hK_V_1.2, hK_V_1.3, hK_Ca_2.1, and hK_Ca_2.2 potassium channels. Representative whole-cell current traces were recorded for (**a**) hK_V_1.1, (**b**) hK_V_1.2, and (**c**) hK_V_1.3 using the voltage protocols shown above the raw current traces every 15 s in the absence (black, control) and presence of 100 nM XYTX_2_-Xa2a (orange) and positive control (TEA^+^ for hK_V_1.1 and hK_V_1.3, and charybdotoxin (ChTx) for hK_V_1.2, blue). (**d**) hK_Ca_2.1 and (**e**) hK_Ca_2.2 currents were elicited with voltage ramps to + 50 mV from a holding potential of − 120 mV every 15 s in the absence (black, control) and presence of XYTX_2_-Xa2a at the indicated concentration (orange) or apamin as a positive control (blue). The currents were corrected for ohmic leakage and then drawn as a function of test potential (E_m_). The horizonal dashed line shows the zero current level, the vertical dashed line indicates the expected reversal potential for K^+^ (− 86.5 mV, based on the Nernst equation). (**f**) Low affinity, concentration-dependent block of hK_Ca_2.2 channels by XYTX_2_-Xa2a. Whole-cell hK_Ca_2.2 currents were recorded using voltage ramps as for (**e**). Remaining current fraction (RCF) was calculated as *I*/*I*_0_ where *I*_0_ is the peak current at + 50 mV in the absence and *I* is the peak current at + 50 mV in the presence of XYTX_2_-Xa2a at equilibrium block at concentrations of 0.1, 1, 5, and 10 μM (empty circles), respectively. Points on the linear dose–response curve represent the mean of 4–6 independent measurements. The line was drawn using linear least squares fit (see Methods for details). The reciprocal of the slope of the best fitted line yielded an IC_50_ of 25.1 ± 3.5 μM. Data are mean ± SEM.

XYTX_2_-Xa2a is similar in sequence to *Bombus* and *Apis* MCD-peptides and apamin. Apamin is a blocker of the mammalian small conductance calcium-activated potassium (K_Ca_, SK) channel K_Ca_2.2^20^, while *Apis* MCD peptide is a blocker of *Shaker*-like voltage-gated potassium (K_V_) channels K_V_1.1 and K_V_1.2^21^. We hypothesised that XYTX_2_-Xa2a might share similar activity. Thus, we tested XYTX_2_-Xa2a for activity on human K_V_1.1, K_V_1.2, K_V_1.3, K_Ca_2.1 and K_Ca_2.2. At a concentration of 100 nM, XYTX_2_-Xa2a did not block K_V_1.1, K_V_1.2 or K_V_1.3 current, but caused a small reversible block of hK_Ca_2.1 and hK_Ca_2.2 current (Fig. 4a–e). We performed a concentration–response experiment for hK_Ca_2.2 (Fig. 4f), where we observed an IC_50_ of 25.1 ± 3.5 μM, with maximal block of ~ 30% at 10 μM. Thus, the pharmacological activity of XYTX_2_-Xa2a appears to be related to that of apamin, although it was less potent and efficacious toward human K_Ca_2.2.

### XYTX_1_-Xa1a and venom PLA_2_ are synergistic pain-causing toxins

*X. aruana* and other venomous Apidae use their venoms exclusively for defense, and many are known to have painful stings^3^. We investigated the contribution of XYTX_1_-Xa1a, XYTX_2_-Xa2a and venom PLA_2_ in this context. For these experiments we used PLA_2_ from *A. mellifera* venom.

**Figure 5:**
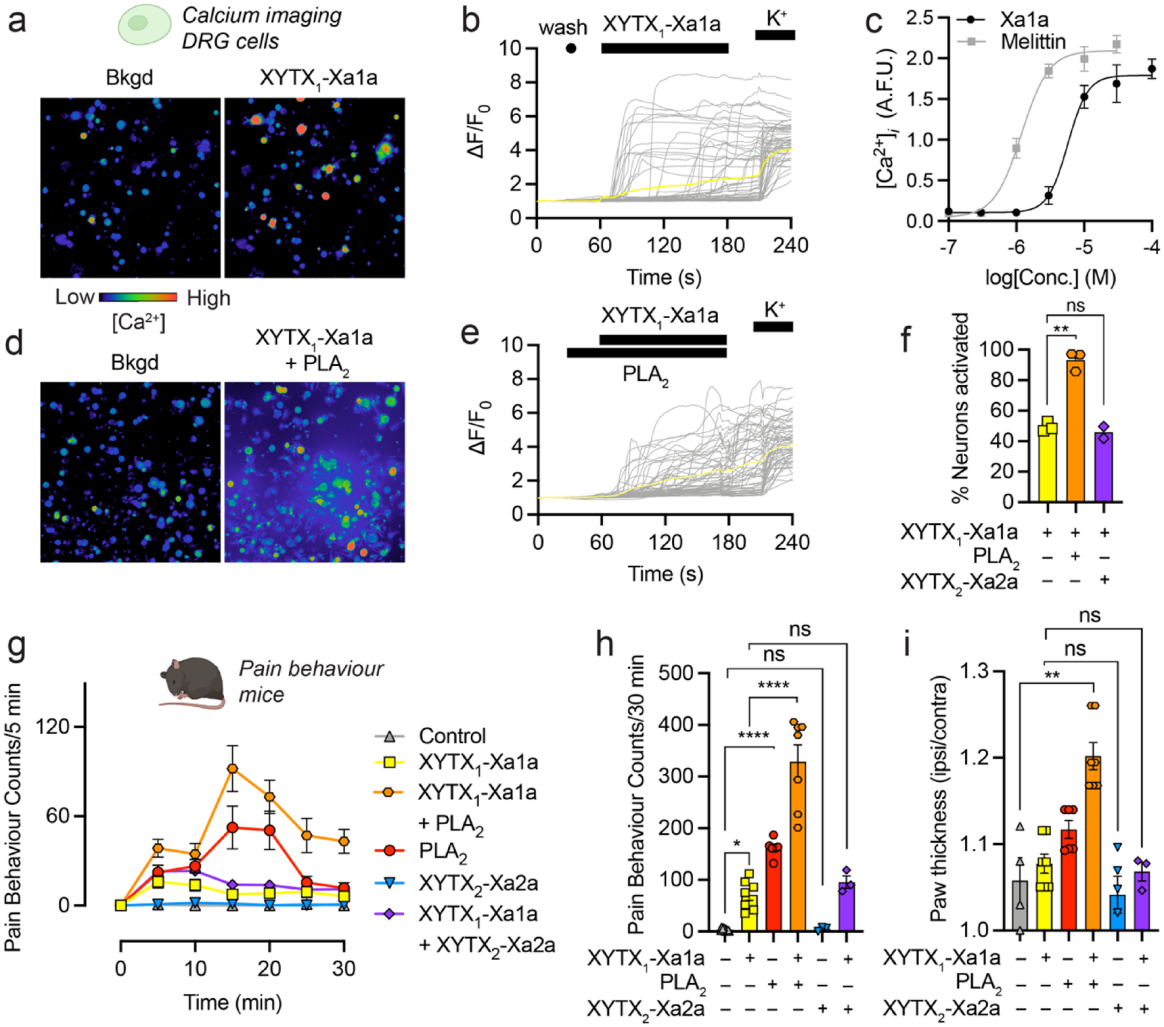
Pain-causing effects of *X. aruana* venom components. (**a,b**) Application of XYTX_1_-Xa1a (10 μM) to DRG cells produced an immediate and sustained, non-cell-specific increase in [Ca^2+^]_*i*_. (**c**) Potency of XYTX_1_-Xa1a and melittin in F11 cells, as monitored by changes in [Ca^2+^]_*i*_. (**d–f**) The increase in [Ca^2+^]_*i*_ caused by XYTX_1_-Xa1a was potentiated by the presence of venom PLA_2_ (1 µM), but not XYTX_2_-Xa2a (1 μM). **P < 0.01 (unpaired *t*-test). (**g,h**) Shallow intraplantar injection of XYTX_1_-Xa1a (200 pmol) caused spontaneous pain behaviours in mice which was potentiated by co-injection of venom PLA_2_ (20 pmol), but not XYTX_2_-Xa2a (20 pmol). XYTX_2_-Xa2a (20 pmol) alone does not cause spontaneous pain behaviours while venom PLA_2_ does (20 pmol). Data are expressed as mean ± SEM (*n* = 3–6). (**i**) Co-injection of XYTX_1_-Xa1a (200 pmol) plus venom PLA_2_ (20 pmol) caused paw swelling. Data are expressed as mean ± SEM (*n* = 3–6). *P < 0.05; **P < 0.01; ****P < 0.0001 (one way-ANOVA with Tukey’s multiple comparisons).

Application of XYTX_1_-Xa1a to cultured mouse dorsal root ganglion (DRG) cells caused an immediate and sustained increase of [Ca^2+^]_*i*_ in 49.4 ± 3.2% of neurons (Fig. 5a–b,f), while application of XYTX_2_-Xa2a or venom PLA_2_ had no direct effect on intracellular calcium levels (Fig. 5d–f). We measured the potency of XYTX_1_-Xa1a in F11 cells (a mouse neuroblastoma × rat DRG cell line) where the peptide caused an increase of [Ca^2+^]_*i*_ with a median effective concentration (EC_50_) of 5.2 ± 0.7 µM (n = 6) (Fig. 5c). In this assay, melittin was more potent (*P* = 0.0002, unpaired t-test; n = 6) with an EC_50_ of 1.2 ± 0.1 µM (n = 6) (Fig. 5c).

Previous studies have shown that *A. mellifera* venom PLA_2_ potentiates the haemolytic activity of melittin^22^, and more recently it was shown that the PLA_2_ toxins of spitting cobra venoms potentiate the nociceptive effects the cobra venom cytotoxins^23^. We hypothesized that the nociceptive effects of XYTX_1_-Xa1a might also be potentiated by PLA_2_. Indeed, activation of DRG neurons by XYTX_1_-Xa1a was increased in the presence of venom PLA_2_ (1 µM) to 93.2 ± 6.5% (*P* = 0.0036, versus XYTX_1_-Xa1a alone, unpaired t-test), which was accompanied by increased cell lysis (Fig. 5d–f). Cell lysis is illustrated in Fig. 5d,e by leakage of dye into the extracellular media. Activation of DRG neurons by XYTX_1_-Xa1a was not increased by the presence of XYTX_2_-Xa2a (45.8 ± 5.5% neurons; *P* = 0.4096, versus XYTX_1_-Xa1a-treated, unpaired t-test) (Fig. 5f).

Shallow intraplantar injection of XYTX_1_-Xa1a (200 pmol) in mice caused spontaneous pain behaviours (sum of pain behaviour counts at 30 min for saline versus XYTX_1_-Xa1a, *P* = 0.0484) (Fig. 5g,h). Injection of 20 pmol XYTX_2_-Xa2a alone did not elicit any spontaneous pain behaviours (compared with negative control, *P* > 0.9999) while injection of venom PLA_2_ alone did (*P* = 0.0001). Consistent with our in vitro data, pain behaviours elicited by XYTX_1_-Xa1a was increased (frequency and duration) by co-injection with 20 pmol venom PLA_2_ (versus XYTX_1_-Xa1a alone, *P* < 0.0001) but not 20 pmol XYTX_2_-Xa2a (*P* = 0.9979). Co-injection of XYTX_1_-Xa1a (200 pmol) and venom PLA_2_ (20 pmol) also caused paw swelling (paw thickness for saline versus XYTX_1_-Xa1a + venom PLA_2_, *P* = 0.0033) (Fig. 5i). These data together with our DRG experiments suggest that PLA_2_ can elicit nociception that is independent of XYTX_1_-Xa1a and immediate activation of DRG neurons—consistent with previous reports of indirect pronociceptive actions of venom PLA_2_s^24,25^—and also synergizes with XYTX_1_-Xa1a to directly activate DRG neurons to cause nociception.

**Figure 6:**
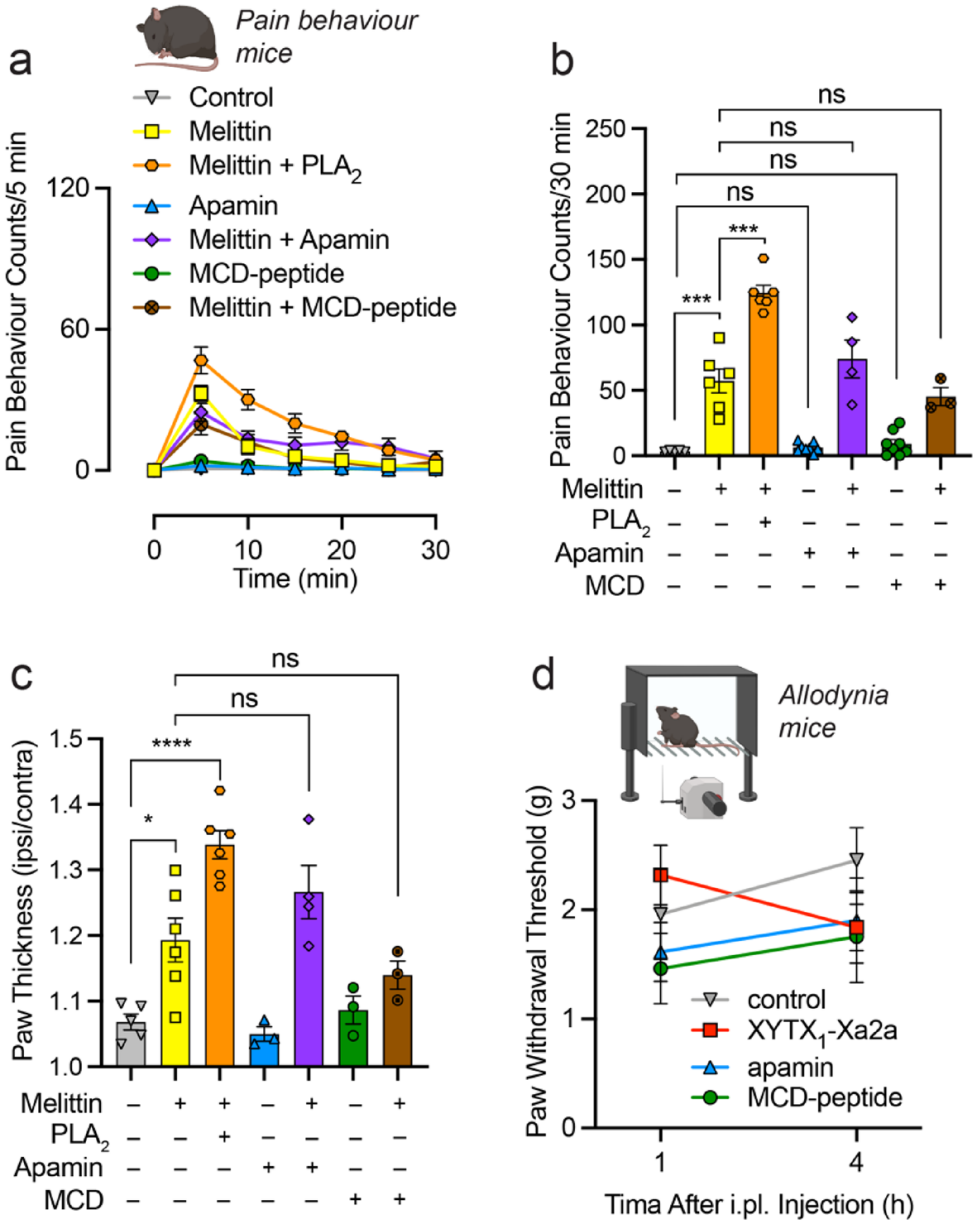
Pain-causing effects of *A. mellifera* venom components. (**a,b**) Shallow intraplantar injection of Melittin (200 pmol) caused spontaneous pain behaviours in mice which was potentiated by co-injection of venom PLA_2_ (20 pmol), but not apamin (2 pmol) or MCD-peptide (2 pmol). Pain behaviour counts elicited by apamin (2 pmol) or MCD-peptide (2 pmol) were not different to control. Data are expressed as mean ± SEM (*n* = 3–8). (**c**) Co-injection of melittin (200 pmol) plus venom PLA_2_ (20 pmol) caused paw swelling. Data are expressed as mean ± SEM (*n* = 3–8). Paw withdrawal thresholds measured by electronic Von Frey, 1 and 4 h after intraplantar injection of XYTX_2_-Xa2a (2 pmol), apamin (2 pmol) or MCD-peptide (2 pmol). ***P < 0.001 ****P < 0.0001; (one-way ANOVA with Tukey’s multiple comparison test).

For comparison, we repeated the aforementioned in vivo experiments with the major venom components of *A. mellifera*; melittin, PLA_2_, apamin and MCD-peptide. Shallow intraplantar injection of mellitin (200 pmol) caused spontaneous pain behaviours (sum of pain behaviour counts at 30 min for saline versus melittin, *P* < 0.0001) (Fig. 6a,b), which was potentiated by co-injection with 20 pmol venom PLA_2_ (versus melittin alone, *P* < 0.0001) but not 2 pmol apamin (*P* = 0.5644) or MCD-peptide (*P* = 0.9801). Neither apamin nor MCD-peptide (2 pmol) alone elicited any spontaneous pain behaviours (compared with negative control, *P* = 0.9998 and 0.9944, respectively). Co-injection of melittin and venom PLA_2_ also caused paw swelling (paw thickness for saline versus melittin + venom PLA_2_, *P* = 0.0010) (Fig. 6c).

These data suggest that XYTX_2_-Xa2a, apamin and MCD-peptide do not contribute to spontaneous pain (in mammals) associated with envenomation by these bees. We therefore tested whether they might contribute to longer-lasting pain responses e.g. allodynia, as has been reported for other hymenopteran venom peptides^26,27^. Using an automated Von Frey apparatus, we measured paw-withdrawal threshold to a mechanical stimulus at 1 and 4 h following intraplantar injection of either XYTX_2_-Xa2a, apamin or MCD-peptide (injected; 2 pmol/paw), where we observed no difference to negative control (saline) injection (Fig. 6d).

## Discussion

In this study, we analysed the venom composition and function of the Australian great carpenter bee *Xylocopa aruana*. While the venom of the eusocial *A. mellifera* is among the most studied of all venoms, there have been few studies on the venoms of solitary Apidae. One probable reason for this is, due to their solitary lifestyle, a greater difficulty in acquiring multiple specimens and therefore sufficient venom and venom-producing tissue for analysis. However as demonstrated here, advances in the sensitivity of mass spectrometry and nucleotide sequencing have now made it possible to analyse the complete venom composition of an individual bee. Working with an individual rather than multiple specimens comes with both advantages and disadvantages: One advantage is that our data were not confounded by intra-specific genetic polymorphisms, which can interfere with transcriptome assembly and conclusions on venom complexity. But this could also be viewed as a potential limitation, i.e. that the venom composition of our specimen may not be an accurate representation of the species. To resolve this comprehensively would require the individual analysis of multiple specimens. However we note that studies of the congeneric *X. appendiculata*^13^ and *X. violacea*^14^ which used multiple individuals, present what appear to be near identical venom compositions to what we report here for *X. aruana*. This suggests that intra-specific variation in venom composition in *X. aruana* is unlikely to alter the major conclusions of our study.

Solitary and eusocial Apidae contend with distinct defensive selection pressures i.e. *Xylocopa* spp. sting solely in self-defence, while the eusocial *A. mellifera* stings in both self-defence *and* defence of its colony, including against large vertebrates. Defensive adaptations in the eusocial Apidae include alarm pheromones, increased aggression and sting autotomy (in *Apis*), which likely serve to increase the *dose* of venom that can be delivered to an aggressor. One might expect that the differing selection pressures between *Apis* and *Xylocopa* would also be reflected in differences in venom composition. However, our data suggest that *X. aruana* and *A. mellifera* share a similar venom composition and venom mode-of action. The sole difference we observed was in the potency of the major pain-causing components—melittin is approximately fivefold more potent than XYTX_1_-Xa1a at activating mammalian sensory neurons. We speculate that the greater potency of melittin, from the eusocial *A. mellifera* venom, over XYTX_1_-Xa1a may represent an additional adaptation in response to the distinct defensive selection pressures associated with the transition to eusociality. While this is consistent with a previous report of a correlation between venom lethal capacity and colony weight in stinging hymenopterans^19^, broader taxon sampling within the Apidae and in other bees, and comparative evaluation of venom potency, will be valuable in further testing this hypothesis.

In both *X. aruana* and *A. mellifera*, one amphipathic pore-forming peptide is the major venom component and the primary pain-causing agent. Venom PLA_2_ increases the pain-causing effects of the amphipathic peptide. Such toxin synergy is widely believed to occur in venoms, yet very few examples have been documented to-date. Other examples include the toxin “cabals” of cone snail venoms, where several toxins with complementary activity work together to achieve rapid paralysis of the prey^28^. Similarly PLA_2_ in cobra venoms potentiates the pain-causing activity of venom cytotoxins^23^. The potentiation, by venom PLA_2_, of the pain-causing effects of melittin and the melittin-like peptide XYTX_1_-Xa1a, in the venoms of *A. mellifera* and *X. aruana*, respectively, is a third example of toxin synergy. We found that bee venom PLA_2_ also induced pain behaviours in its own right, and thus can also be considered a pain-causing agent. We did not resolve the mechanism by which this occurs, although it appears to be independent of direct activation of sensory neurons. In contrast to the other major venom components, the apamin-like peptides, which are blockers of potassium channels and make up the final major class of polypeptides in these venoms, did not cause spontaneous pain behaviours or allodynia and their contribution(s) in the context of defence and pain remains unclear. This study contributes to our understanding of the evolution, chemistry and pharmacology of the venoms of the Apidae.

## Methods

### Venom collection

A single adult female *X. aruana* specimen was collected with permission of the landholders at Aroona station, Queensland, Australia. The venom apparatus was dissected in PBS. Venom glands were placed directly in RNAlater and stored at − 20 °C, and venom was squeezed from the dissected venom reservoir and venom duct into 20 μL water and stored at − 20 °C. The total amount of venom collected from the individual, estimated from A_280_ measured using a Nanodrop spectrophotometer (Thermo Fisher, Waltham, MA, USA), was 142 μg.

### Transcriptome sequencing and assembly

Using TRIzol (Life Technologies, Carlsbad, CA, USA), total RNA (148 ng) was extracted from the venom glands. Complementary DNA library preparation and sequencing was performed by the UQ Institute for Molecular Bioscience Sequencing Facility. A dual-indexed library was constructed with the TruSeq-3 Stranded mRNA Sample Prep Kit (Illumina, San Diego, CA, USA) with oligo (dT) selection and an average insert size of 180 base pairs. The sample was pooled in a batch of 20 samples, and 150-cycle paired-end sequencing was performed on an Illumina NextSeq 500 instrument. Adapter trimming of demultiplexed raw reads was performed using fqtrim v0.9.7^29^, followed by quality trimming and filtering using prinseq-lite v0.20.4^30^. Error correction was performed using BBnorm tadpole, part of the BBtools package. Trimmed and error-corrected reads were assembled using Trinity v2.4.0^31^ with a k-mer length of 31 and a minimum k-mer coverage of 2. Assembled transcripts were annotated using a BLASTX^32^ search (E value setting of 1e^−3^) against the UniRef90 database. Estimates of transcript abundance were performed using the RSEM^33^ plugin of Trinity (align_and_estimate_abundance). Using TransDecoder, Transcripts were translated and filtered to open-reading frames (> 30 amino acid residues). This was used as a search database for ProteinPilot.

### Mass spectrometry

A combination of top-down proteomics of native and reduced and alkylated venom, and bottom-up proteomics of reduced, alkylated and trypsin-digested venom was used to examine the venom composition of the individual *X. aruana*. Two aliquots of venom (10 μg each) were dried by vacuum centrifugation. Gas phase reduction and alkylation was performed according to the protocol described by Hale et al.^34^. 100 μL of reduction/alkylation reagent (50% (v/v) ammonium carbonate, 48.75% ACN, 1% 2-iodoethanol, 0.25% triethylphosphine was added to the lid of each 1.5 mL tube containing dried venom, which was then inverted, closed, and incubated at 37 °C for 90 min. One aliquot of reduced and alkylated venom was then digested by incubating with trypsin (20 ng/μL) overnight at 37 °C, according to the manufacturer’s instructions (Sigma-Aldrich, St. Louis, MO, USA).

Three venom samples (10 μg each)—native venom, reduced and alkylated venom, and reduced, alkylated and trypsin-digested venom—were analyzed by LC–MS/MS. Samples were separated on a Nexera uHPLC (Shimadzu, Kyoto, Japan) with a Zorbax stable-bond C18 column (2.1 × 100 mm; particle size, 1.8 μm; pore size, 300 Å; Agilent, Santa Clara, CA, USA), using a flow rate of 180 μL/min and a gradient of 1–40% solvent B (90% ACN and 0.1% formic acid (FA)) in 0.1% FA over 25 min, 40–80% solvent B over 4 min, and analyzed on an AB Sciex 5600 TripleTOF (SCIEX, Framingham, MA, USA; operated with Analyst TTF v1.8) mass spectrometer equipped with a Turbo-V source heated to 550 °C. MS survey scans were acquired at 300 to 1800 mass/charge ratio (m/z) over 250 ms, and the 20 most intense ions with a charge of + 2 to + 5 and an intensity of at least 120 counts were selected for MS/MS. The unit mass precursor ion inclusion window mass within 0.7 Da and isotopes within 2 Da were excluded from MS/MS, with scans acquired at 80 to 1400 m/z over 100 ms and optimized for high resolution. Using ProteinPilot v5.0 (SCIEX), MS/MS spectra were searched against the translated venom apparatus transcriptome (MS and MS/MS tolerance of 0.05 and 0.1 Da, respectively). False discovery rate analyses were generated by ProteinPilot default method, which uses a decoy database.

Transcripts encoding venom components were then manually examined using the Map-to-Reference tool of Geneious v10.2.6^35^, where two paralogues of XYTX_1_-Xa1a were reassembled. These were then reincorporated back into the complete transcriptome, estimation of transcript abundance repeated, and a second, final ProteinPilot search performed. Peptides identified by ProteinPilot were validated by comparison of experimentally derived MS/MS peaks against a theoretical peak list generated using MS-Product in ProteinProspector v5.22.1 (http://prospector.ucsf.edu/prospector/cgi-bin/msform.cgi?form=msproduct).

For MALDI-TOF MS, 0.7 µg of venom was spotted together with 0.5 µL α-cyanohydroxycinnamic acid (CHCA) and allowed to dry, then analyzed using a SCIEX 5800 MALDI-TOF MS in reflectron-positive mode with laser power 4400–5000. The m/z range was 1000–5000 m/z.

### Peptide synthesis

Peptides were produced using Fmoc solid-phase peptide synthesis at 0.1 mmol scale. Protecting groups used were Lys/Trp/His(Boc), Ser/Thr/Tyr(tBu), Asp/Glu(OtBu), Asn/Gln/Cys/His(Trt), and Arg(Pbf). Peptides were assembled on Rink-amide ProTide resin (CEM, Matthews, NC) on a CEM Liberty Prime HT24 microwave synthesizer (CEM Corp, Matthews, NC, USA) using N,N'-diisopropylcarbodiimide (DIC)/oxyma and Fmoc groups were removed with 20% pyrrolidine, as per manufacturers protocols.

Peptides were released from resin by treatment with 95% TFA/2.5% H_2_O/2.5% trisiopropylsilane (TIPS). Peptides were precipitated with 15 mL ice-cold ether, extracted in A/B 50/50 (A: 0.05% TFA, B: 90% ACN, 0.045% TFA) and lyophilized prior to purification. Peptides were purified on a Shimadzu Prominence LC-20AT RP-HPLC system equipped with a SPD-20AV UV detector and a FRC-10A fraction collector using a Gemini C18 column (2.1 × 200 mm; particle size, 5 μm; pore size, 110 Å; Phenomenex, Torrence, CA, USA) at 8 mL/min. Gradients used were 0–60% B over 60 min (XYTX_1_-Xa1a) and 0–50% B over 50 min (XYTX_2_-Xa2a). Fractions of interest were lyophilized and purity assessed using ESI MS and analytical RP-HPLC. XYTX_2_-Xa2a was oxidized at 0.1 mg/mL in 1 M NH_4_OAc at pH 8.0 overnight at room temperature and purified and analyzed as described above.

LC–MS was used to compare the elution times of oxidised synthetic XYTX_2_-Xa2a and native XYTX_2_-Xa2a in the venom. 10 μg native venom was separated on a Nexera uHPLC with a Zorbax stable-bond C18 column, using a flow rate of 180 μL/min and a gradient of 1–40% solvent B (90% ACN and 0.05% TFA) over 18 min and analyzed on an AB Sciex 5600 TripleTOF mass spectrometer. 1 nmol oxidised synthetic XYTX_2_-Xa2a (red) was analysed under the same conditions. The elution time of the extracted ion chromatogram (XIC) of 629.0627 ± 0.05 m/z (theoretical (M + 4H)^4+^ ion of XYTX_2_-Xa2a) was compared.

Melittin and bee venom PLA_2_ were purchased from Sigma-Aldrich (St. Louis, MO, USA), and apamin and MCD-peptide were purchased from Alomone labs (Jerusalem, Israel).

### Whole cell voltage-clamp electrophysiology

HEK293AD cells (American Type Culture Collection) were cultured as previously described^36^. Cells were maintained on DMEM supplemented with 10% heat-inactivated FBS, 2 mM l-glutamine, pyridoxine and 110 mg/mL sodium pyruvate. Whole-cell patch-clamp experiments were performed using a QPatch 16X automated electrophysiology platform (Sophion Bioscience). The extracellular solution contained the following: 70 mM NaCl, 70 mM choline chloride, 4 mM KCl, 2 mM CaCl_2_, 1 mM MgCl_2_, 10 mM Hepes, and 10 mM glucose (pH 7.4 with NaOH; 305 mosmol). The intracellular solution contained the following: 140 mM CsF, 1 mM:5 mM EGTA/CsOH, 10 mM Hepes, and 10 mM NaCl (pH 7.3 with CsOH; 320 mosmol). From a holding potential of 0 mV each recorded cell was subjected to a series of 50-ms voltage pulses that ranged from − 60 to + 60 mV in 10-mV increments. Recordings were made prior to and 4 min after the addition of either ECS (negative control) or XYTX_1_-Xa1a (10 µM). Data are mean ± SEM of 5–6 experiments and fitted to a simple linear regression.

Chinese Hamster Ovarian (CHO) cells (American Type Culture Collection) were grown in DMEM-high glucose supplemented with 10% FBS, 2 mM l-glutamine, 100 U/mL penicillin-g, and 100 μg/mL streptomycin (Invitrogen) at 37 °C in a 5% CO_2_ and 95% air humidified atmosphere. Cells were passaged twice per week following a 7-min incubation in PBS containing 0.2 g EDTA/L (Invitrogen). hK_V_1.1, hK_V_1.2, hK_Ca_2.1, and hK_Ca_2.2 channels were transiently expressed in CHO cells using Lipofectamine 2000 (Invitrogen Carlsbad, CA), following the manufacturer’s protocol and were cultured under standard conditions. For recording hK_V_1.1, hK_V_1.2, and hK_Ca_2.1 currents GFP-tagged ion channel vectors were used. The hK_Ca_2.2 channel plasmid was transiently co-transfected with a plasmid encoding the green fluorescent protein (GFP) at a molar ratio of 1:10. Transfected cells were washed twice with 2 mL of ECS (see below) and replated onto 35-mm polystyrene cell culture dishes (Cellstar, Greiner Bio-One). Currents were recorded 24 to 48 h after transfection. GFP-positive transfectants were identified with a Nikon Eclipse TS100 fluorescence microscope (Nikon, Tokyo, Japan) using bandpass filters of 455–495 nm and 515–555 nm for excitation and emission, respectively and were used for current recordings (> 70% success rate for co-transfection). hK_V_1.3 currents were recorded on activated lymphocytes 3 to 4 days after activation. The human veinous blood was obtained from anonymized healthy donors. The peripheral blood mononuclear cells were isolated by Histopaque1077 (Sigma-Aldrich Hungary, Budapest, Hungary) density gradient centrifugation. Cells obtained were resuspended in RPMI 1640 medium containing 10% fetal calf serum (FCS, Sigma-Aldrich), 100 μg/mL penicillin, 100 μg/mL streptomycin, and 2 mM l-glutamine, seeded in a 24-well culture plate at a density of 5–6 × 10^5^ cells/mL, and grown in a 5% CO_2_ incubator at 37 °C for 3–5 days. Phytohemagglutinin A (Sigma-Aldrich) was added in 10 μg/mL concentrations to the medium to amplify the K_V_1.3 expression. Cells were washed gently twice with 2 mL of ECS (see below) for the patch-clamp experiments. Standard whole-cell patch-clamp method^37^ was used to record ionic currents. Micropipettes were pulled in four stages by using a Flaming Brown automatic pipette puller (Sutter Instruments, San Rafael, CA) from Borosilicate Standard Wall with Filament aluminum–silicate glass (Harvard Apparatus Co., Holliston, MA) with tip diameters between 0.5 and 1 μm and heat polished to a tip resistance ranging typically 2–8 MΩ. All measurements were carried out by using Axopatch 200B amplifier connected to a personal computer using Axon Digidata 1550A data acquisition hardware, respectively (Molecular Devices Inc., Sunnyvale, CA). In general, the holding potential was − 120 mV. Records were discarded when leak at the holding potential was more than 10% of the peak current at the test potential. Experiments were done at room temperature ranging between 20 and 24 °C. Data were analysed using GraphPad Prism 8 (Graphpad, CA, USA) and pClamp10.5 software package (Molecular Devices Inc., Sunnyvale, CA). Before analysis, whole-cell current traces were corrected for ohmic leakage and were digitally filtered with a three-point boxcar smoothing filter. For hK_Ca_2.1–2 the reversal potential for K^+^ was determined and only those currents were analyzed for which the reversal potential fell into the range of the theoretical reversal potential ± 5 mV (− 86.5 ± 5 mV). For hK_V_1.1, hK_V_1.2, and hK_V_1.3, the extracellular (bath) solution (ECS) contained 145 mM NaCl, 5 mM KCl, 2.5 mM CaCl_2_, 1 mM MgCl_2_, 10 mM Hepes, and 5.5 mM glucose (pH 7.35 with NaOH, 302–308 mOsM), and the intracellular (pipette) solution (ICS) contained 140 mM KF, 2 mM MgCl_2_, 1 mM CaCl_2_, 11 mM EGTA, and 10 mM Hepes (pH 7.2 with KOH, ~ 295 mOsM). For hK_Ca_2.1 and hK_Ca_2.2, the extracellular solution (ECS) contained 145 mM l-Aspartic acid with Na, 5 mM KCl, 2.5 mM CaCl_2_, 1 mM MgCl_2_, 10 mM Hepes, and 5.5 mM glucose (pH 7.4 with NaOH), and the intracellular solution (ICS) contained 145 mM K-Asp, 2 mM MgCl_2_, 8.5 mM CaCl_2_, 10 mM EGTA, and 10 mM Hepes (pH 7.2 with KOH) giving ~ 2 µM free Ca^2+^ to fully activate the hK_Ca_2.1–2 currents. XYTX_2_-Xa2a (and the positive controls) were dissolved in the ECS supplemented with 0.1 mg/mL BSA (Bovine Serum Albumin). Bath perfusion around the measured cell with different extracellular solutions was achieved using a gravity flow micro perfusion system at a rate of 0.5 mL/min. Excess fluid was removed continuously. For measurements of currents on hK_V_1.1–3 voltage steps to + 50 mV were applied from a holding potential of − 120 mV every 15 s and the peak current was measured. hK_Ca_2.1–2 currents were elicited every 15 s with voltage ramps to + 50 mV from a holding potential of − 120 mV. The remaining current fraction (RCF) at a given molar concentration was calculated as *I/I*_0_, where *I*_0_ is the peak current at + 50 mV in the absence and *I* is the peak current at + 50 mV in the presence of XYTX_2_-Xa2a at equilibrium block at a given concentration, respectively. The data points on dose–response curve represent the mean of 4–6 individual measurements and fitted with a simple linear regression according to the equation of Y = 1 + slope × [toxin], where Y is the reciprocal of the remaining current fraction (1/RCF) and [toxin] is the molar concentration of XYTX_2_-Xa2a. The reciprocal of the slope yielded IC_50_.

### Animal ethics

All experiments involving animals or animal tissues were approved by the University of Queensland animal ethics committee (approval numbers PHARM/526/18, 2021/AE000448), were conducted in accordance with local and national regulations including the *International Association for the Study of Pain Guidelines for the Use of Animals in Research* in agreement with the *Animal Care and Protection Regulation Qld* (2012), and the *Australian Code of Practice for the Care and Use of Animals for Scientific Purposes, 8th edition* (2013) and followed the recommendations in the ARRIVE guidelines.

### Calcium imaging assay of mouse DRG neurons and F11 cells

DRG cells were isolated from 4- to 6-week-old-male C57BL/6 mice that were purchased from the Animal Resources Centre (WA, Australia). DRGs were dissociated, then cells were plated in Dulbecco’s Modified Eagle’s Medium (DMEM; Gibco, MD, USA) containing 10% fetal bovine serum (FBS) (Assaymatrix, VIC, Australia) and penicillin/streptomycin (Gibco) on a 96-well poly-d-lysine-coated culture plate (Corning, ME, USA) and maintained overnight. Cells were loaded with Fluo-4 AM calcium indicator, according to the manufacturer’s instructions (ThermoFisher Scientific, MA, USA). After loading (1 h), the dye-containing solution was replaced with assay solution (1× Hanks’ balanced salt solution, 20 mM HEPES). Images were acquired at 10× objective at 1 frame/s (excitation 485 nm, emission 521 nm). Fluorescence corresponding to [Ca^2+^]_*i*_ of ~ 250 cells per experiment was monitored in parallel using an Nikon Ti-E Deconvolution inverted microscope, equipped with a Lumencor Spectra LED Lightsource. Baseline fluorescence was monitored for 30 s. At 30 s, assay solution was replaced with either assay solution, or assay solution containing venom PLA_2_ (1 µM) or XYTX_2_-Xa2a (1 µM), then at 1 min with XYTX_1_-Xa1a (in assay solution ± venom PLA_2_ (1 µM) or XYTX_2_-Xa2a (1 µM)) and monitored for 2 min before being replaced with assay solution and then KCl (30 mM; positive control). Experiments involving the use of mouse tissue were approved by the University of Queensland Animal Ethics Committee (UQ AEC; approval number TRI/IMB/093/17).

F11 (mouse neuroblastoma $\times$ DRG neuron hybrid; European Collection of Authenticated Cell Cultures) were cultured as previously described^36^. Cells were maintained on Ham’s F12 media supplemented with 10% FBS, 100 µM hypoxanthine, 0.4 µM aminopterin, and 16 µM thymidine (Hybri-Max, Sigma Aldrich). 384-well imaging plates (Corning, Lowell, MA, USA) were seeded 24 h prior to calcium imaging, resulting in ~ 90% confluence at the time of imaging. Cells were loaded for 30 min at 37 °C with Calcium 4 assay component A in physiological salt solution (PSS; 140 mM NaCl, 11.5 mM d-glucose, 5.9 mM KCl, 1.4 mM MgCl_2_, 1.2 mM NaH_2_PO_4_, 5 mM NaHCO_3_, 1.8 mM CaCl_2_, 10 mM HEPES) according to the manufacturer’s instructions (Molecular Devices, Sunnyvale, CA). Ca^2+^ responses were measured using a FLIPR^TETRA^ fluorescent plate reader equipped with a CCD camera (Ex: 470 to 490 nm, Em: 515 to 575 nM) (Molecular Devices, Sunnyvale, CA). Signals were read every second for 10 s before, and 300 s after, the addition of peptide (in PSS supplemented with 0.1% BSA).

### Pain behaviour experiments

Male adult (6 weeks old) C57BL/6J mice were used for behavioral experiments. To facilitate injections mice were briefly anesthetized using 2.5% isoflurane. Each peptide diluted in saline containing 0.1% bovine serum albumin (BSA), was administered in a volume of 20 µL into the hind paw by shallow intraplantar injection. Negative control animals were injected with saline containing 0.1% BSA. Following injection, spontaneous pain behaviour events were counted from video recordings by a researcher blinded to the treatments. Mechanical paw withdrawal thresholds were measured 1 and 4 h following injection using automated Von Frey apparatus (MouseMet; Topcat Metrology).

### Statistics

Data were plotted and analysed using Prism v9.0.0 (GraphPad Software, San Diego, CA, USA). For calcium imaging experiments of mouse DRG neurons and F11 cells, treatment groups were compared using unpaired *t*-tests. For analysis of spontaneous pain, sum of pain behaviour counts at 30 min of treatment groups were compared using one-way ANOVA with Tukey’s multiple comparisons test. Statistical significance was defined as *P* < 0.05. All data are presented as mean ± SEM.

## Supplementary Information

Supplementary Figures.

Supplementary Information.

## Data Availability

Prepropeptide sequences of XYTX_1_-Xa1a, XYTX_2_-Xa2a and the *X. aruana* venom PLA_2_ have been deposited with GenBank, under accessions: ON586842, ON586843 and ON5868424, respectively. RNA-seq reads have been deposited in the NCBI sequence read archive under accessions SRR22306546. The mass spectrometry proteomics data have been deposited to the ProteomeXchange Consortium via the PRIDE^38^ partner repository with the dataset identifier PXD038183.
