# Supplementary material for: Venom composition and pain-causing toxins of the Australian great carpenter bee Xylocopa aruana
Source: Sci Rep. 2022 Dec 22;12:22168. doi: 10.1038/s41598-022-26867-8 (PMC9780326; doi:10.1038/s41598-022-26867-8)
Supplement: Supplementary file 1 — Supplementary Figures. [file 41598_2022_26867_MOESM1_ESM.docx]

**Supplementary Information:**

**
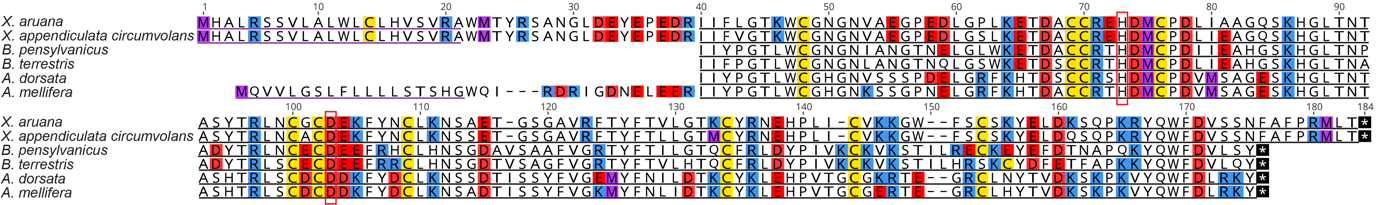
 Figure S1. Amino acid sequence alignments of the PLA_2_ toxins of *X. aruana* venom with homologues from other Apidae venoms.** PLA_2_ from the venom of *X. aruana*, *X. appendiculata* (Uniprot: I7GQA7), *B. pensylvanicus* (Uniprot: Q7M4I6), *B. terrestris* (Uniprot: P82971), *A. dorsata* (Uniprot: Q7M4I5) and *A. mellifera* (Uniprot: P00630). Methionine, lysine/arginine, aspartate/glutamate, and cysteine residues and stop codons are highlighted in purple, blue, red, yellow and black respectively. Signal peptides and mature peptides are underlined in purple and grey, respectively. Active site residues are boxed in red, post-translational modifications are not shown.


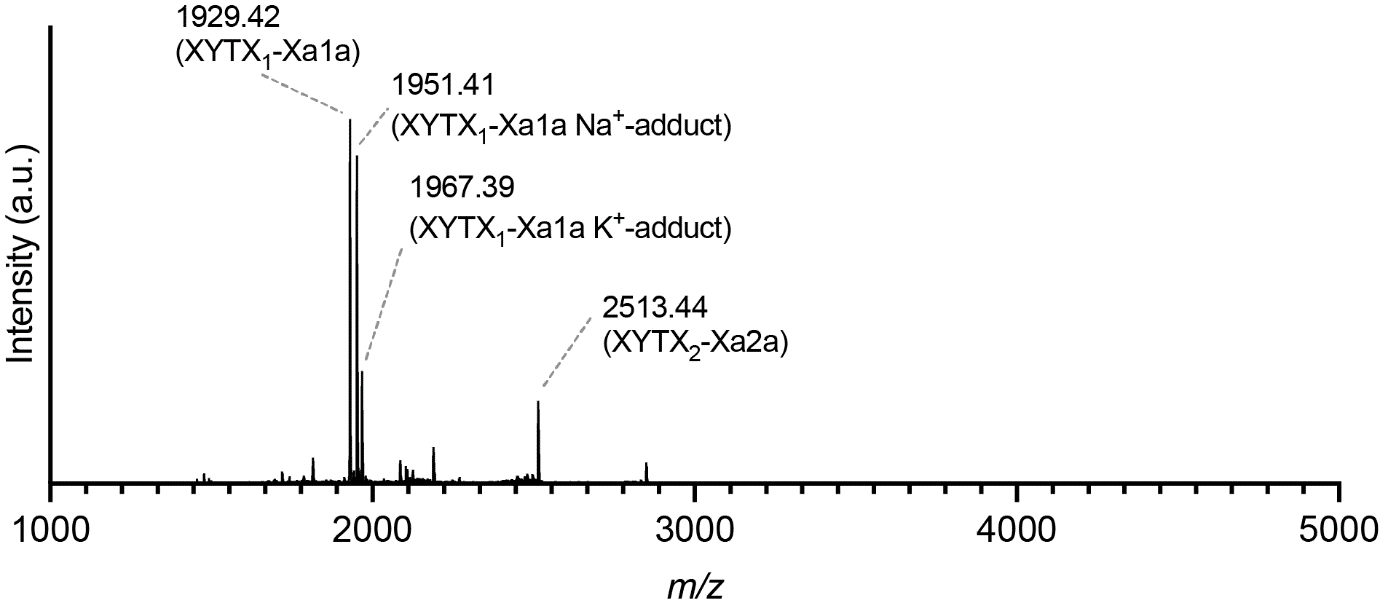


**Figure S2. MALDI-TOF mass spectra obtained in reflectron positive mode of *X. aruana* venom.** The four main peaks are labelled with the experimental [M+H]^+1^ monoisotopic masses. Theoretical [M+H]^+1^ monoisotopic masses are as follows: XYTX_1_-Xa1a = 1929.28; XYTX_2_-Xa2a = 2513.22.


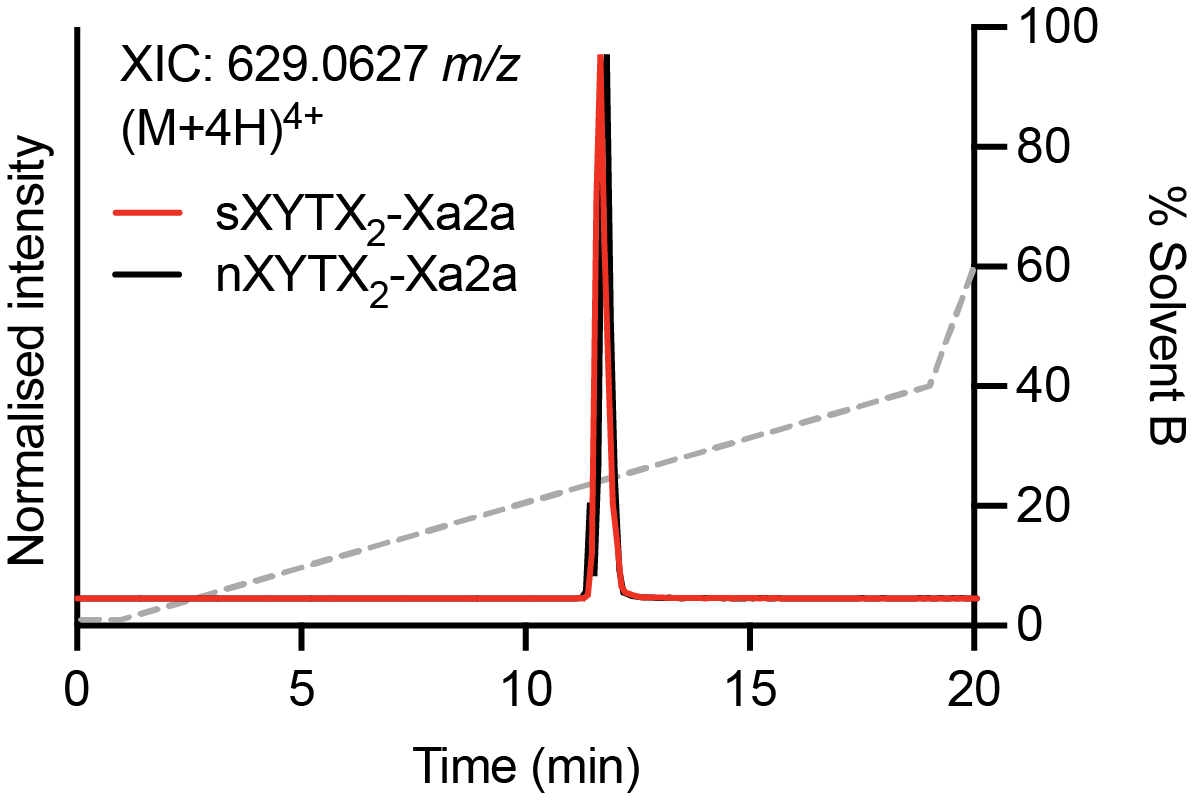


**Figure S3. Oxidised synthetic XYTX_2_-Xa2a elutes at the same retention time as native XYTX_2_-Xa2a in the venom.** Extracted ion chromatogram (XIC) of 629.0627 ± 0.05 m/z (theoretical (M+4H)^4+^ ion of XYTX_2_-Xa2a) from LC-MS/MS of 10 μg native venom (black) and 1 nmol oxidised synthetic XYTX_2_-Xa2a (red) using a gradient of 1–40% solvent B (90% acetonitrile (ACN) and 0.05% trifluoroacetic acid (TFA)) over 18 min.


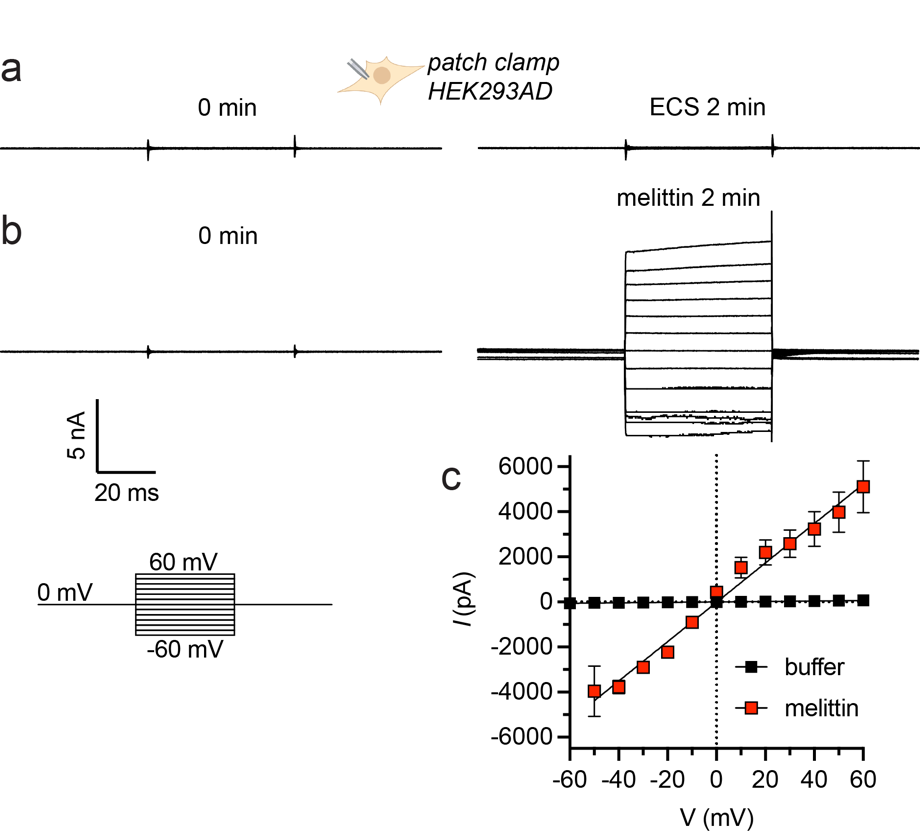


**Figure S4. Melittin causes a leak current in HEK293AD cells.** (a) Representative current traces in HEK293AD cells at voltages of –60 to +60 mV (10 mV steps) acquired before application and 2 min after application of (a) extracellular solution (ECS) or (b) 10 μM melittin. Scale bar for panels a-b and voltage protocol used to investigate leak current are shown on the bottom left. (c) Current-voltage (*I*-V) relationship 2 min after addition of ECS (black) or melittin (10 μM; yellow). Data are expressed as mean ± SEM (*n* = 5 cells) and fitted to a simple linear regression.
